# An autoencoder-based deep learning method for genotype imputation

**DOI:** 10.3389/frai.2022.1028978

**Published:** 2022-11-03

**Authors:** Meng Song, Jonathan Greenbaum, Joseph Luttrell, Weihua Zhou, Chong Wu, Zhe Luo, Chuan Qiu, Lan Juan Zhao, Kuan-Jui Su, Qing Tian, Hui Shen, Huixiao Hong, Ping Gong, Xinghua Shi, Hong-Wen Deng, Chaoyang Zhang

**Affiliations:** ^1^School of Computing Sciences and Computer Engineering, University of Southern Mississippi, Hattiesburg, MS, United States; ^2^Tulane Center of Biomedical Informatics and Genomics, School of Medicine, Tulane University, New Orleans, LA, United States; ^3^College of Computing, Michigan Technological University, Houghton, MI, United States; ^4^Department of Biostatistics, The University of Texas MD Anderson Cancer Center, Houston, TX, United States; ^5^Division of Bioinformatics and Biostatistics, National Center for Toxicological Research, US Food and Drug Administration, Jefferson, AR, United States; ^6^Environmental Laboratory, U.S. Army Engineer Research and Development Center, Vicksburg, MS, United States; ^7^Department of Computer & Information Sciences, Temple University, Philadelphia, PA, United States

**Keywords:** genotype imputation, deep learning, autoencoder, paired sample *t*-test, GWAS

## Abstract

Genotype imputation has a wide range of applications in genome-wide association study (GWAS), including increasing the statistical power of association tests, discovering trait-associated loci in meta-analyses, and prioritizing causal variants with fine-mapping. In recent years, deep learning (DL) based methods, such as sparse convolutional denoising autoencoder (SCDA), have been developed for genotype imputation. However, it remains a challenging task to optimize the learning process in DL-based methods to achieve high imputation accuracy. To address this challenge, we have developed a convolutional autoencoder (AE) model for genotype imputation and implemented a customized training loop by modifying the training process with a single batch loss rather than the average loss over batches. This modified AE imputation model was evaluated using a yeast dataset, the human leukocyte antigen (HLA) data from the 1,000 Genomes Project (1KGP), and our in-house genotype data from the Louisiana Osteoporosis Study (LOS). Our modified AE imputation model has achieved comparable or better performance than the existing SCDA model in terms of evaluation metrics such as the concordance rate (CR), the Hellinger score, the scaled Euclidean norm (SEN) score, and the imputation quality score (IQS) in all three datasets. Taking the imputation results from the HLA data as an example, the AE model achieved an average CR of 0.9468 and 0.9459, Hellinger score of 0.9765 and 0.9518, SEN score of 0.9977 and 0.9953, and IQS of 0.9515 and 0.9044 at missing ratios of 10% and 20%, respectively. As for the results of LOS data, it achieved an average CR of 0.9005, Hellinger score of 0.9384, SEN score of 0.9940, and IQS of 0.8681 at the missing ratio of 20%. In summary, our proposed method for genotype imputation has a great potential to increase the statistical power of GWAS and improve downstream post-GWAS analyses.

## Introduction

Genotype imputation has become an essential step in genome-wide association study (GWAS). It is now widely used in a variety of applications in GWAS, such as boosting the power of association studies, increasing the possibility of identifying functional single-nucleotide polymorphisms (SNPs) or causal genetic variants, enhancing the resolution in fine-mapping studies, and discovering trait-associated loci in meta-analyses (Das et al., 2018). Although the cost of whole-genome sequencing (WGS) has decreased considerably during the past few years, it remains cost-prohibitive to perform WGS for a large number of samples. Currently, most GWAS samples are genotyped with low coverage genotyping approaches such as SNP arrays (Torkamaneh and Belzile, 2021). However, these low coverage approaches will inevitably generate incomplete datasets with missing values. Missing values in genotype data can considerably limit causal variants discovery or statistical inferences in meta-analysis. Therefore, it is a necessary step to impute untyped or missing variants before performing association studies.

The first two examples of GWASs facilitated by genotype imputation were a type 2 diabetes (T2D) study in Finns (Scott et al., 2007) and a joint GWAS with 2,000 cases and 3,000 controls from the UK for seven complex diseases such as coronary artery disease (CAD) and T2D (Burton et al., 2007). From then on, genotype imputation has become an important step in GWAS for human disease studies. Another recent example is a meta-analysis with 44,506 samples to identify genomic risk loci for bone mineral density (BMD) in an osteoporosis study (Greenbaum et al., 2022). For five independent GWAS array samples in this study, missing values were imputed by using Minimac2 (Fuchsberger et al., 2015, 2) with the Trans-Omics for Precision Medicine (TOPMed) (including > 97,000 high coverage genomes with a mean depth of 30×) as a reference panel.

The presence of missing values in SNP genotyping arrays is a common issue and can have various causes, such as assay failures, the design of different densities for genotyping platforms, and the detection of rare variants. Current genotype imputation methods can be divided into two classes: reference-based and reference-free approaches. The reference-based genotype imputation methods need a large-scale reference panel such as TOPMed and the assumption behind them is that individuals from the same or similar ancestor can share short stretches of DNA sequence between them (Song et al., 2020). Therefore, the observed genotypes from an SNP array can be used to match DNA segments shared between a target sample with missing values and a reference panel without missing values. Reference-based imputation methods include IMPUTE5 (Rubinacci et al., 2020), BEAGLE5 (Browning et al., 2018), Minimac4 (Das et al., 2016), MACH (Li et al., 2010), and fastPHASE (Scheet and Stephens, 2006). In recent years, web-based imputation tools appeared, such as the TOPMed Imputation Server (https://imputation.biodatacatalyst.nhlbi.nih.gov/), the Michigan Imputation Server (https://imputationserver.sph.umich.edu/), and the Sanger Imputation server (https://www.sanger.ac.uk/tool/sanger-imputation-service/). However, there are some challenges for these reference-based methods such as the computational cost of genotype calling for a large number of samples in a reference panel and the restrictive nature of obtaining consent for general research use (Das et al., 2018).

In contrast, reference-free imputation methods such as mean replacement, singular value decomposition (SVD), k-nearest neighbors (KNN), and random forest (RF) do not require a reference panel. In recent years, deep learning (DL) has had a great impact on many application areas, such as natural language processing, image processing, and bioinformatics because of its ability to accommodate large datasets and model highly non-linear relationships. By combining autoencoder (AE) and convolutional neural networks (CNNs), a reference-free approach, SCDA (Sparse Convolutional Denoising Autoencoder), was used for genotype imputation (Chen and Shi, 2019). It utilizes the advantages of convolutional layers to extract local data correlations within nearby variants in an AE model structure. However, the SCDA model was implemented sequentially, and has some limitations such as the inability to handle shared information with another layer except for its subsequent layer as well as the inability to build a model with multiple inputs and outputs. In addition, the training process for the SCDA model is based on minimizing a default average loss over batches and researchers are not able to implement a custom training loop, which may be needed to further improve the performance. Therefore, there is a need to modify the model and its implementation to improve the performance of genotype imputation.

In this paper, we present an improved one-dimensional (1D) convolutional AE model, inspired by SCDA, to perform genotype imputation. Instead of using sequential or functional methods to define the neural network architectures, we utilized the model subclassing method to build our AE model as it can be more easily extended to other omics data (e.g., gene expression data). Compared with sequential and functional methods, our model subclassing method is fully customizable and enables researchers to have control over every detail of the deep neural network and the whole training pipeline. With these advantages of the model subclassing method, we improved the training process by implementing a customized training loop and using a single batch loss. We evaluated our modified AE model with two public genotype datasets [yeast data and the human leukocyte antigen (HLA) data in the 1,000 Genomes Project (1KGP)] and our own genotype data generated from the Louisiana Osteoporosis Study (LOS) project. Compared with the SCDA model, our AE model achieved a comparable or better concordance rate (CR), Hellinger score, scaled Euclidean norm (SEN) score, and imputation quality score (IQS).

## Materials and methods

### Dataset sources and data preprocessing

We used three genotype datasets in this study, including the yeast data (Chen and Shi, 2019), HLA data (Chen and Shi, 2019), and LOS data (Greenbaum et al., 2022). We selected the first two publicly available datasets to benchmark the performance of our imputation approach. Then we applied our model to the LOS data, which was recently collected at Tulane University and aimed to investigate the molecular mechanisms of osteoporosis by integrating multi-omics data.

#### Yeast data

The yeast genotype data (Bloom et al., 2015; Chen and Shi, 2019) from the SCDA model has 28,220 variants from 4,390 samples. There are two strains of yeast: an isolate from a vineyard (RM) encoded with −1 and a laboratory strain (BY) encoded with 1, respectively. We replaced all RM variants of −1 with 2 to make sure that there were no negative values when calculating the categorical cross entropy (CCE) loss function for the model.

#### HLA data

The aim of the 1KGP was to provide researchers with a comprehensive open data source of human genetic variation by using technologies such as microarray genotyping, low coverage WGS with a mean depth of 7.4×, and deep exome sequencing with a mean depth of 65.7× (Auton et al., 2015; Zheng-Bradley and Flicek, 2017). The phase 3 of 1KGP (released in 2005) included 2,504 individuals from 26 multiple populations. Given the high quality of genotype data from 1KGP, it can serve as a reference panel for reference-based genotype imputation methods such as IMPUTE5 (Rubinacci et al., 2020) and BEAGLE5 (Browning et al., 2018). Specifically, HLA genes from the major histocompatibility complex (MHC) region at 6p21.3 are considered to contribute to a wide range of complex human diseases (Naito et al., 2021) and the genotypes in this HLA region are more diverse and heterogeneous (Chen and Shi, 2019). The HLA region from the 1KGP contains 28,583 genotypes from 2,504 individuals across five populations including Americans, Europeans, Africans, East Asians, and Southern Asians (Auton et al., 2015; Chen and Shi, 2019). After removing multi-allelic SNPs with Bcftools for the HLA data, there were 27,209 SNPs remaining across 2,504 individuals that are used in this study.

#### LOS data

LOS is an ongoing research study that has recruited >17,000 individuals since 2011 and aims to investigate the genetic risk factors of osteoporosis and other complex diseases (Greenbaum et al., 2022). Table 1 shows a summary of the gender and ethnicity for the available subjects in the LOS data until June 2022. In total, there are 4,986 unrelated subjects including 2,110 African Americans and 2,876 Caucasians randomly selected (stratified by sex and race groups) from the whole LOS cohort. WGS of the blood samples was conducted on a BGISEQ-500 sequencer (BGI Americas Corporation, Cambridge, MA, USA) with 350 bp paired-end reads at an average sequencing depth of 22× (Greenbaum et al., 2022). By using the Burrows-Wheeler Aligner software, sequence reads were aligned to the human reference genome (version GRCh38/hg38) (Li and Durbin, 2009). Single-nucleotide variants (SNVs) and small Insertion–deletion mutations (InDels) were detected with the HaplotypeCaller of the Genome Analysis Toolkit (GATK) (McKenna et al., 2010). Variant quality score recalibration (VQSR) was applied to filter out potential sequencing artifacts and obtain high confidence variant calls (McKenna et al., 2010).

**Table 1:** Distribution of LOS samples based on gender and ethnicity.

**Gender/**	**African**	**Caucasian**	**Total**
**ethnicity**	**American**		
Male	1,124	1,357	2,481
Female	986	1,519	2,505
Total	2,110	2,876	4,986

**Figure 1:**
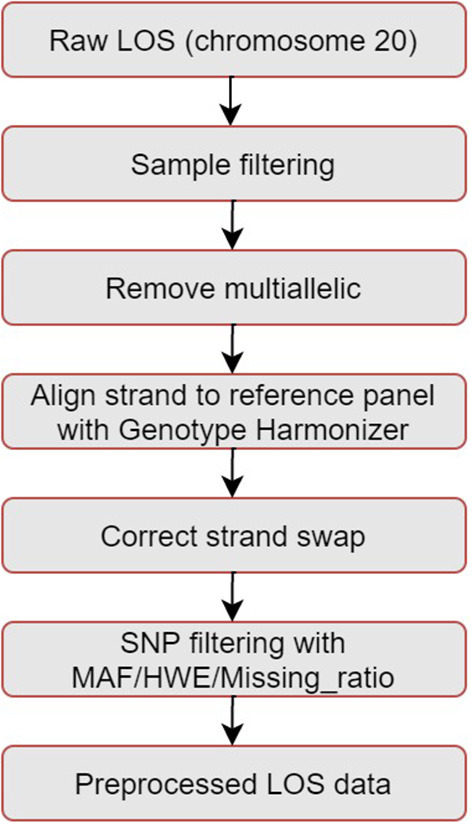
Pipeline of quality control for the LOS data. Taking chromosome 20 as an example, it has 3,098,112 SNVs from 4,986 samples. After quality control, we retained 162,027 SNPs from 4,985 samples.

**Figure 2:**
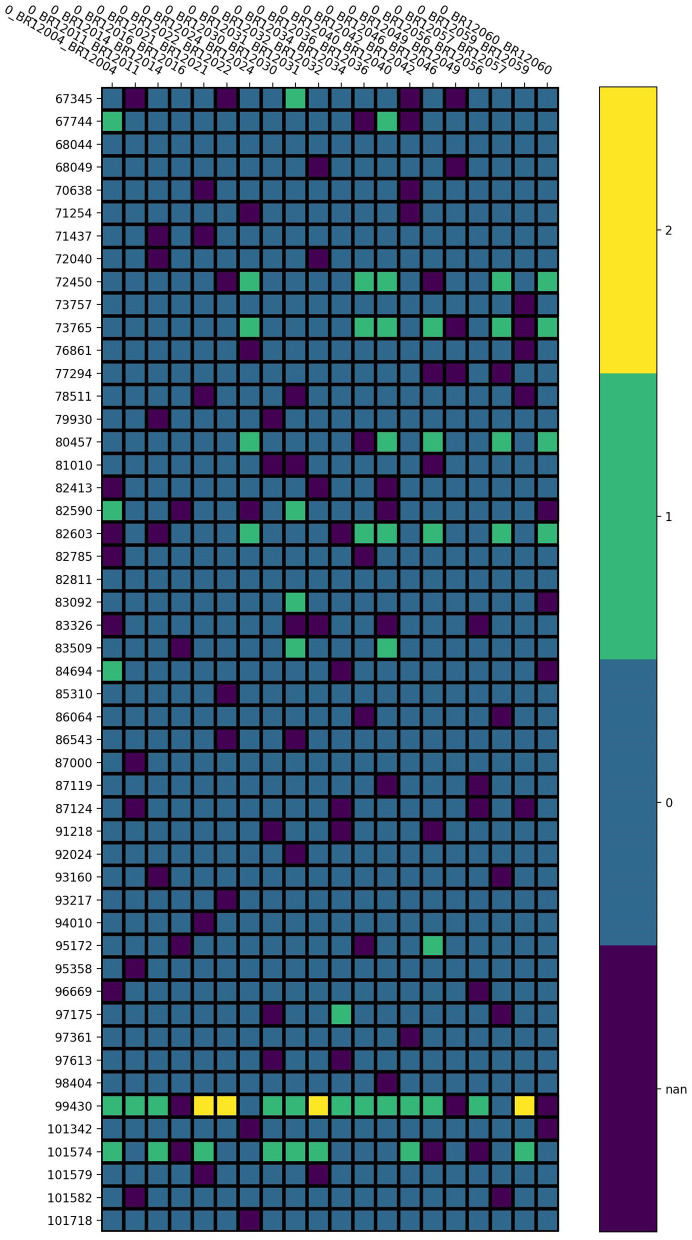
Visualization of the preprocessed LOS data (chromosome 20) with a heatmap at the missing ratio of 10%. Rows represent the position of each SNP and columns indicate different samples. Different colors represent different genotype values: purple for missing value, blue for 0, green for 1, and yellow for 2.

The pipeline for quality control of the LOS genotype data is illustrated in Figure 1. Taking chromosome 20 as an example, it has 3,098,112 SNVs from 4,986 samples. We first performed sample filtering with PLINK (Purcell et al., 2007) to exclude samples with more than 95% of the genotype missing. We then used Bcftools to remove multiallelic variants (Danecek et al., 2021). To solve the unknown strand issue, we aligned the strands of genotype data to the latest version of 1KGP reference panel with a mean depth of 30× (GRCh38/hg38) (Aganezov et al., 2022) by using Genotype Harmonizer (GH) (Deelen et al., 2014). GH automatically aligns ambiguous A/T and G/C SNPs to the reference by using linkage disequilibrium (LD) patterns without prior knowledge of the strands. Next, we corrected the strand swaps with the fixref library of Bcftools and excluded any remaining unmatched SNPs for the reference genome with “Bcftools norm -check-ref x” (Danecek et al., 2021). Then, we used Vcftools (Danecek et al., 2011) to perform SNP filtering with the following criteria: missing ratio > 0% (removing all missing values), Hardy-Weinberg equilibrium (HWE) *p*-value < 10^−6^, and minor allele frequency (MAF) < 0.1%. After the above quality control steps, we retained 162,027 SNPs from 4,985 samples for chromosome 20 in LOS data. Figure 2 visualizes a subset of the preprocessed LOS data (chromosome 20) with a heatmap at the missing ratio of 10%.

#### One-hot encoding of preprocessed data

Table 2 shows a summary of the three datasets after preprocessing. For the encoding of the HLA and LOS data, we first added one to all the original genotype values of 0 (0|0), 1 (0|1 or 1|0) and 2 (1|1), which represents the number of non-reference alleles. Therefore, the corresponding new genotype values are 1, 2, and 3, respectively. The purpose for doing this is to use zeros to represent fake missing values for evaluating the imputation performance (Chen and Shi, 2019). Then, we utilized one-hot encoding for these genotype values with 0 encoded as (1,0,0,0), 1 as (0,1,0,0), 2 as (0,0,1,0), and 3 as (0,0,0,1). As for the yeast data, we conducted similar processing procedures with 0 encoded as (1,0,0), 1 as (0,1,0), and 2 as (0,0,1).

**Table 2:** Summary of three genotype datasets after preprocessing.

**Data**	**Samples**	**Number of SNPs**
Yeast	4,390	28,220
HLA	2,504	27,209
LOS (chromosome 20)	4,985	162,027

In addition, to determine the impact of minor allele frequency (MAF) on the imputation accuracy for the preprocessed LOS data (chromosome 20), we divided the SNPs into four groups according to their MAFs (as shown in Table 3): MAF > 5% (38,872 SNPs), 1% < MAF < 5% (59,579 SNPs), 0.5% < MAF < 1% (33,899 SNPs), and 0.1% < MAF < 0.5% (29,677 SNPs). The thresholds of missingness and HWE for the quality control remain the same.

**Table 3:** SNPs of LOS (chromosome 20) data with different MAFs.

**MAF**	**Number of SNPs**
MAF > 5%	38,872
1% < MAF < 5%	59,579
0.5% < MAF < 1%	33,899
0.1% < MAF < 0.5%	29,677

### AE model architecture

An AE is an unsupervised artificial neural network that learns a low-dimensional latent space representation from high-dimensional input data and then reconstructs the output data from the learned representation (Goodfellow et al., 2016). It consists of two components: an encoder and a decoder. The structure of an AE can be defined as:


(1) $$\begin{array}{l} \hat{x}\;=D\left(E\left(x\right)\right) \end{array}$$

where *x* is the input, $\hat{x}$ is the output, E is the encoder sub-network of the AE, and D is the decoder sub-network of the AE. The decoder usually has an inverted symmetric structure to the encoder. The number of nodes for the stacked layers in the encoder usually decreases while the number of nodes for the decoder increases back to the number of the AE's input. The loss function for an AE can be defined as:


(2) $$\begin{array}{l} L\left(x,\hat{x}\right) \end{array}$$

Among the different types of AE structures, a denoising AE receives corrupted data by injecting some noise into the original input and predict the uncorrupted output. If we corrupt the input genotype data with some missing values, the denoising AE is able to recover these missing values for genotype imputation. On the other hand, CNNs have been widely used for two-dimensional image classification problems. Similarly, they can be applied to 1D genotype data with one-hot encoding for human data. The equation for a 1D CNN can be described as follows (González-Muñiz et al., 2020):


(3) $$\begin{array}{l} x_{l}^{\left(m\right)}=\delta \left(b_{l}^{\left(m\right)}+\overset{C}{\underset{c=1}{\sum }}W_{l}^{\left(c,m\right)}*x_{l-1}^{\left(c\right)}\right) \end{array}$$

where (^*^) is the convolution operator between the input $x_{l-1}^{\left(c\right)}$ and the weight of the *m-*th filter $W_{l}^{\left(c,m\right)}$ at the *c-*th channel, *C* denotes the number of channels, *l* represents the number of layers, $b_{l}^{\left(m\right)}$ is the bias for the *m-*th filter at layer *l*, δ is the activation function (such as rectified linear unit (ReLU) and sigmoid), and $x_{l}^{\left(m\right)}$ is the output.

**Figure 3:**
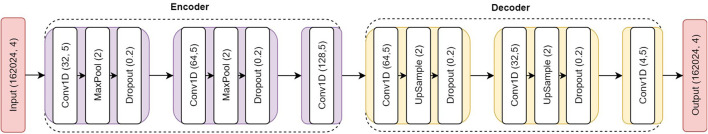
Overview of the AE structure for genotype imputation. The input is the fake missing SNPs with one-hot encoding and the output is the probability of the imputed SNPs. The six consecutive 1D convolutional layers have 32, 64, 128, 64, 32, and 4 filters, respectively. All of them have a filter size of five and L1 regularization factor of 0.0001.

We implemented a 1D convolutional AE model to perform genotype imputation (Figure 3). Taking the LOS data (chromosome 20) as an example, we selected the first 162,024 SNPs out of the total 162,027 SNPs according to their positions to ensure that the number of SNPs is divisible by 4. Here the number 4 is determined by the product of the number of convolutional layers (e.g., 2) of the encoder and the pool size (e.g., 2). The input SNPs data have been converted from two dimensions (4,985 samples, 162,024 SNPs) into three dimensions (4,985 samples, 162,024 SNPs, 4 channels) with one-hot encoding. The first two 1D convolutional layers for the SNP encoder have 32 and 64 filters, respectively. Each of them is followed by a max-pooling layer (with a pool size of 2) and a dropout (with a dropout rate of 0.2) layer. The embedding layer of the AE model is a 1D convolutional layer with 40,506 features and 128 filters. The SNP decoder has an inverted symmetry structure with the encoder. The first two 1D convolutional layers for the SNP decoder have 64 and 32 filters, respectively. Each of them is followed by an up-sampling layer (with a factor of 2) and a dropout (with a dropout rate of 0.2) layer. Finally, we used a 1D convolutional layer with 162,024 features, 4 channels, and the “Softmax” activation function as the output layer. All the convolutional layers of the AE model have a filter size of five and each of them has an L1 regularization factor of 0.0001.

### Loss function

The loss function of the AE model can be defined as a reconstruction error between the input and imputed output such as CCE for discrete values or mean squared error (MSE) for continuous values. Since genotype data are discrete values, we utilized CCE as the loss function:


(4) $$\begin{array}{l} L_{CCE}\left(x,\;\hat{x}\right)=-\frac{1}{N}\overset{N}{\underset{i=0}{\sum }}\overset{C}{\underset{j=0}{\sum }}x_{ij}*\log\left({\hat{x}}_{ij}\right) \end{array}$$

where N is the total number of data points (i.e., the product of the number of samples and the number of SNPs), C is the number of channels, *x*_*ij*_ is the input SNP with one-hot encoding for the *i-*th sample of the *j*-th channel, and ${\hat{x}}_{ij}$ is the probability of imputed SNP. Then we defined a weighted CCE to train the AE model:


(5) $$\begin{array}{l} L\;\left(x,\;\hat{x}\right)=\alpha L_{CCE}\left(x,\;\hat{x}\right) \end{array}$$

where α is the weight of CCE loss. In our AE model, we set α as 1.

### SCDA model for baseline comparison

The SCDA model is based on a general denoising AE framework for genotype imputation (Chen and Shi, 2019). To capture the LD patterns among nearby genetic markers, it utilizes the CNNs in an AE structure. In total, the SCDA model has six convolutional layers with the number of filters set as 32, 64, 128, 128, 64, and 1, respectively. The size of all the filters is 5 × 1 and an L1 regularization (λ = 0.0001) was introduced to each convolutional layer to add a sparsity constraint for the high dimensional genotype data. Two max-pooling layers with a pool size of 2 were deployed in the encoder network to reduce the dimension of the input features, whereas two up-sampling layers with a factor of 2 were used in the decoder network to restore the dimension for the imputed output features. In addition, the SCDA model uses dropout layers (with a rate of 0.25) to prevent overfitting. For the input genotype data, it uses the one-hot encoding technique. The loss function for the model is CCE.

### Model training strategy

We implemented the proposed AE model with TensorFlow v2.4.1. We utilized the model subclassing method in the Keras framework to implement our AE model as it is more flexible and can be easily extended to other omics data (e.g., gene expression). At the same time, the model subclassing method offers us the opportunity to have full control of the model. Thus, it enables us to implement a custom training loop and improve the training process by using a single batch loss rather than the average loss over batches.

We first divided the preprocessed genotype data into training, validation, and test data by randomly splitting the samples with the proportion of 64%, 16%, and 20%, respectively. Next, to compare the performances between our AE model and the SCDA model, we generated three datasets by randomly masking with enforced missing rates of 0%, 10%, and 20% after data splitting. This process replaced random values in the original genotype datasets with zeros to create missing values for each of the preprocessed genotype datasets including yeast, HLA, and LOS. A summary of the hyperparameter settings for our AE model is shown in Table 4. For example, we set the batch size as 32 and the number of epochs as 100. During the training process, we used the Adam optimizer with an initial learning rate of 0.001.

**Table 4:** Summary of the hyperparameter settings for our AE model.

**Hyperparameters**	**Values**
Epochs	100
Batch size	32
Initial learning rate	0.001
Dropout	0.2
L1 regularization	0.0001
Max-pooling size	2
Up-sampling size	2
Filter size	5
Number of filters	(32, 64, 128, 64, 32, 4)
Strides	1
Padding	Same
Optimizer	Adam
Activation function	ReLU except the output layer (Softmax)

### Evaluation metrics

We evaluated our AE model in terms of the evaluation metrics CR, Hellinger score, SEN score, and IQS for all the experiments as well as the Pearson correlation coefficient (PCC) in the LOS genotype imputation experiment (Stahl et al., 2021). The CR is the ratio of correctly imputed SNPs out of all SNPs. The Hellinger score is a measure of the distance between two probability distributions, while the SEN score is the scaled Euclidean distance between the true dosage (the expectations of the observed distribution) and the imputed dosage (the expectations of the imputed posterior distribution) (Roshyara et al., 2014). Both the Hellinger score and SEN score are calculated per SNP and per sample. The IQS is calculated based on the observed proportion of agreement and the chance agreement (Lin et al., 2010). The details for the definition of the equations for these metrics are shown in Supplementary material.

The above four evaluation metrics were based on the comparison between imputed genotypes and the ground truth of the sequenced genotypes (Stahl et al., 2021). For the calculation of the CR, we first calculated the values across SNPs for each sample, and then determined the mean value for all samples. As for the IQS, we first calculated the values across samples for each SNP, and then obtained the mean values for all SNPs. Since the Hellinger score and SEN score are calculated per SNP and per sample, we needed to accumulate them (e.g., the mean and the minimum) across samples for each SNP, and then determine the mean values for all SNPs. The minimum of the Hellinger score and the minimum of the SEN score can be viewed as the lower bound of the imputation quality. The range of all the evaluation metrics is from 0 to 1, and a score close to 1 indicates a higher imputation quality.

A paired sample *t*-test (Ross and Willson, 2017) was used to compare the evaluation metrics between our AE model and the SCDA model and to determine if there is a significant difference between them. Since we selected the same random seed for data splitting on both models and ensured the same test samples for each comparison, we chose to perform a paired *t*-test rather than a standard two sample *t*-test for comparing the mean evaluation metrics between the two models.

### Experimental setup

We trained and tested the AE model and then compared it with the SCDA model on both our Seahawk server and the Tulane BIZON HPC server. Seahawk consists of four NVIDIA GP102 Titan X (Pascal) GPUs, an Intel Xeon CPU E5 1650 V4, and 98 GB system memory. The Tulane BIZON HPC server consists of two NVIDIA RTX A6000 48 GB GPUs, an AMD Ryzen Threadripper 3970X CPU, and 128 GB DDR4 system memory.

## Results

To evaluate the performance of our AE model, we compared it to the SCDA method with three different genotype datasets including yeast, HLA, and LOS. We chose the CR, the Hellinger score, the SEN score, and the IQS as evaluation metrics and calculated the average value and standard deviation (SD) as well as the corresponding *p*-value by running the models three times at three different missing ratios (0%, 10%, and 20%). In addition, we visualized the results of evaluation metrics between these two models with violin plots and histograms. Lastly, we assessed the impact of MAFs on the imputation quality with the LOS data.

### Impacts of the improved training processes

**Figure 4:**
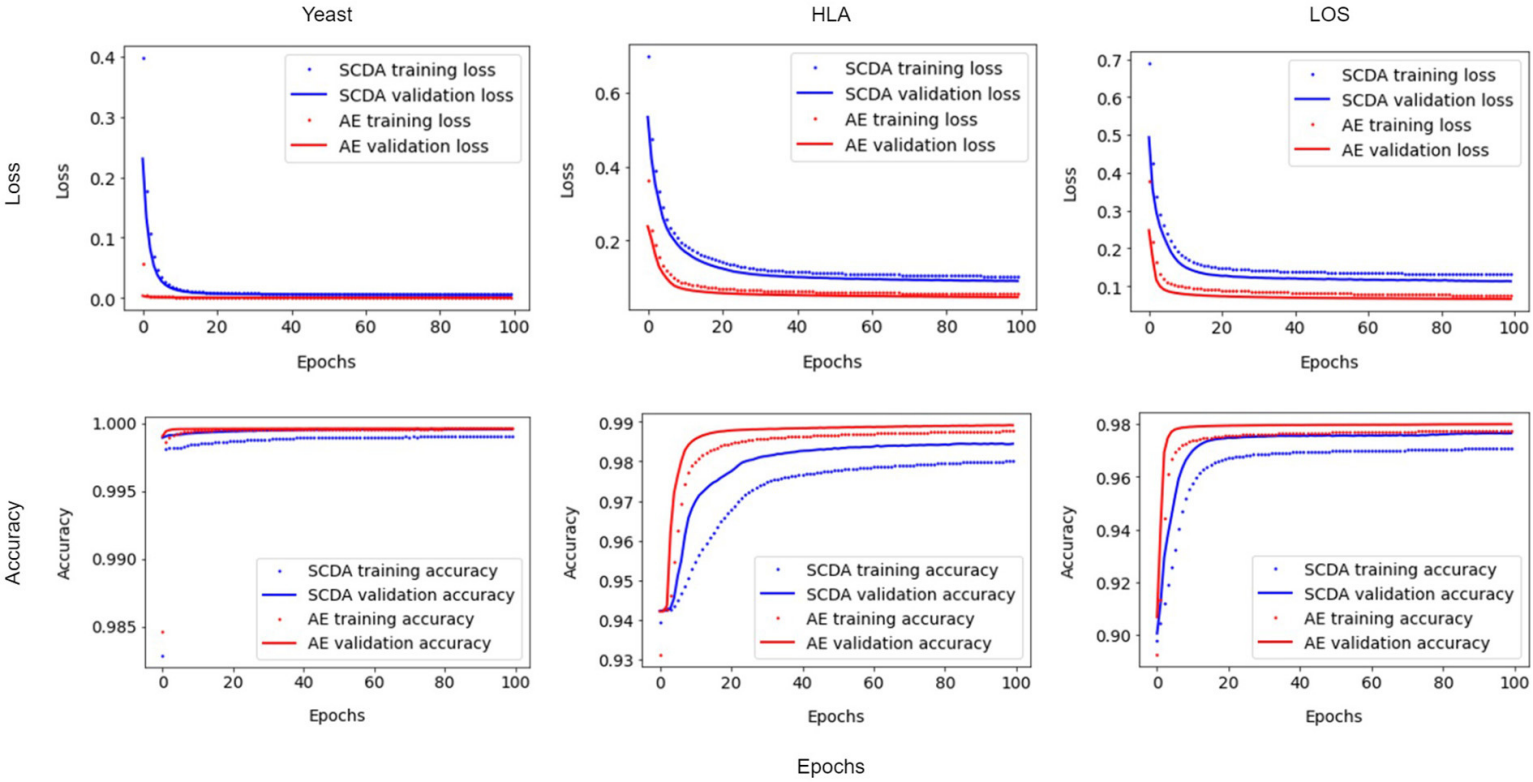
Comparison of loss **(Top)** and accuracy **(Bottom)** curve between the SCDA (blue color) and the AE (red color) model during training (dotted line) and validation (solid line) processes on the yeast, HLA, and LOS data (from left to right) at the missing ratio of 20%.

We implemented a customized training loop and modified the training process by using a single batch loss rather than the running average loss over batches. Since the results of running the training process between our AE model and the SCDA model over three attempts were very similar, we chose the first instance as an example. Figure 4 shows the improvements for the loss and accuracy curve of our AE model compared to the SCDA model during training and validation processes on three different training and validation datasets, especially for the HLA and LOS genotype data, at the missing ratio of 20%. The results show that our AE model converges faster than the SCDA model and achieves comparable or higher accuracy than the SCDA model for both training and validation processes.

### Imputation performance comparison

Table 5 shows the performance comparison of evaluation metrics between our AE model and the SCDA model on three test datasets at different missing ratios. We observed that our AE model achieved overall better or comparable imputation performance than the SCDA model in all metrics.

**Table 5:** Performance results (mean, SD, and *p*-value with respect to different evaluation metrics) between the AE and the SCDA model on three different test datasets at different missing ratios (0%, 10% and 20%).

**Metrics**	**Data**	**Model**	**Missing ratio**					
			**0%**		**10%**		**20%**	
			**mean (SD)**	***p*** **-value**	**mean (SD)**	***p*** **-value**	**mean (SD)**	***p*** **-value**
CR (accuracy)	Yeast	SCDA	0.9999 (0.0)	0.0090	0.9979 (0.0)	0.0422	0.9970 (0.0)	0.4710
		AE	1.0000 (0.0)		0.9980 (0.0)		0.9979 (0.0)	
	HLA	SCDA	0.9947 (0.0008)	0.0103	0.9421 (0.0003)	0.0019	0.9416 (0.0002)	0.0014
		AE	1.0000 (0.0)		0.9468 (0.0001)		0.9459 (0.0001)	
	LOS	SCDA	0.9983 (0.0003)	0.0141	0.9005 (0.0007)	0.0393	0.8999 (0.0007)	0.0166
		AE	0.9999 (0.0)		0.9011 (0.0006)		0.9005 (0.0007)	
Hellinger score	Yeast	SCDA	0.9979 (0.0007)	0.0540	0.9974 (0.0005)	0.0312	0.9963 (0.0006)	0.0240
		AE	1.0000 (0.0)		0.9995 (0.0001)		0.9991 (0.0)	
	HLA	SCDA	0.9843 (0.0016)	0.0056	0.9506 (0.0010)	0.0004	0.9192 (0.0017)	0.0001
		AE	0.9995 (0.0)		0.9765 (0.0005)		0.9518 (0.0012)	
	LOS	SCDA	0.9880 (0.0010)	0.0037	0.9404 (0.0028)	0.0048	0.9043 (0.0007)	0.0004
		AE	0.9993 (0.0001)		0.9687 (0.0001)		0.9384 (0.0005)	
Minimum Hellinger score	Yeast	SCDA	0.7180 (0.0049)	0.0002	0.6477 (0.0056)	0.0006	0.6151 (0.0076)	0.0017
		AE	0.9765 (0.0004)		0.8389 (0.0018)		0.7551 (0.0020)	
	HLA	SCDA	0.8245 (0.0157)	0.0047	0.5596 (0.0118)	0.1395	0.5022 (0.0092)	0.0391
		AE	0.9826 (0.0003)		0.5753 (0.0042)		0.5119 (0.0072)	
	LOS	SCDA	0.7058 (0.0175)	0.0021	0.1626 (0.0015)	0.0054	0.1049 (0.0019)	0.0002
		AE	0.9471 (0.0048)		0.2079 (0.0033)		0.1413 (0.0012)	
SEN score	Yeast	SCDA	1.0000 (0.0)	0.0007	0.9999 (0.0)	0.0018	0.9999 (0.0)	0.0070
		AE	1.0000 (0.0)		1.0000 (0.0)		0.9999 (0.0)	
	HLA	SCDA	0.9981 (0.0003)	0.0130	0.9952 (0.0004)	0.0156	0.9929 (0.0002)	0.0014
		AE	1.0000 (0.0)		0.9977 (0.0)		0.9953 (0.0001)	
	LOS	SCDA	0.9995 (0.0001)	0.0227	0.9958 (0.0)	0.0003	0.9925 (0.0)	0.0014
		AE	1.0000 (0.0)		0.9970 (0.0)		0.9940 (0.0)	
Minimum SEN score	Yeast	SCDA	0.9795 (0.0003)	0.0001	0.9636 (0.0007)	0.0010	0.9526 (0.0013)	0.0038
		AE	0.9976 (0.0001)		0.9821 (0.0001)		0.9700 (0.0002)	
	HLA	SCDA	0.9702 (0.0043)	0.0106	0.8436 (0.0036)	0.0027	0.8195 (0.0048)	0.0045
		AE	0.9991 (0.0001)		0.8591 (0.0026)		0.8301 (0.0041)	
	LOS	SCDA	0.9681 (0.0062)	0.0188	0.6389 (0.0007)	0.0019	0.5751 (0.0035)	0.0015
		AE	0.9976 (0.0006)		0.6719 (0.0025)		0.6059 (0.0019)	
IQS	Yeast	SCDA	0.9998 (0.0)	0.0090	0.9993 (0.0)	0.0001	0.9990 (0.0)	0.0107
		AE	1.0000 (0.0)		0.9996 (0.0)		0.9991 (0.0)	
	HLA	SCDA	0.9604 (0.0056)	0.0099	0.9115 (0.0067)	0.0129	0.8678 (0.0057)	0.0113
		AE	0.9996 (0.0001)		0.9515 (0.0002)		0.9044 (0.0022)	
	LOS	SCDA	0.9899 (0.0040)	0.0730	0.9145 (0.0023)	0.0064	0.8470 (0.0023)	0.0079
		AE	0.9997 (0.0001)		0.9355 (0.0003)		0.8681 (0.0005)	

First, for the yeast data, our AE model achieved slightly better or at least comparable performance than the SCDA model in terms of the evaluation metrics CR, Hellinger score, SEN score, and IQS. Both models achieved almost the same performance regardless of the missing ratios. In contrast, for the minimum of the Hellinger score and the minimum of the SEN score, our model achieved considerably better results than the SCDA model on the data with three different missing ratios. The performance of these two metrics for both models declined with increasing missing ratios. Second, for the HLA data, our AE model had better performance than the SCDA model in all of the metrics at three different missing ratios, except one case of the minimum of the Hellinger score with the missing ratio of 10%, which shows no significant difference based on the paired sample *t*-test (*p*-value = 0.1395). On the other hand, even though the imputation performances for both models declined when the missing ratios increased, our AE model still outperformed the SCDA model. Finally, for the LOS data, our AE model performed better than the SCDA model in all the metrics at three different missing ratios except one case of the IQS with the missing ratio of 0% showing no significant improvement (*p*-value = 0.0730). Although the performance of both models decreased with the increase of missing ratios, our AE model yielded better performance than the SCDA model.

To test if there is a significant improvement between our AE model and the SCDA model, we calculated the mean and SD of each metric as well as the corresponding *p*-values (Table 5). We observed that most of the *p*-values were below a significance level of 0.05, which indicated a significant improvement between our AE model and the SCDA model.

We noticed that the imputation performance on yeast genotype data was much better than that on human genotype datasets including HLA and LOS data with different missing ratios, especially with high missing ratios (e.g., 20%). As discussed in the SCDA paper (Chen and Shi, 2019), the correlation patterns among nearby genetic markers in yeast genotype data are considerably stronger than those among human genotype data, which led to a higher imputation performance with the yeast data. Compared with yeast, human genotypes are highly dispersed and heterogeneous, leading to more difficulty for the human genotype imputation than for yeast data.

#### Visualization of metrics with violin plots

**Figure 5:**
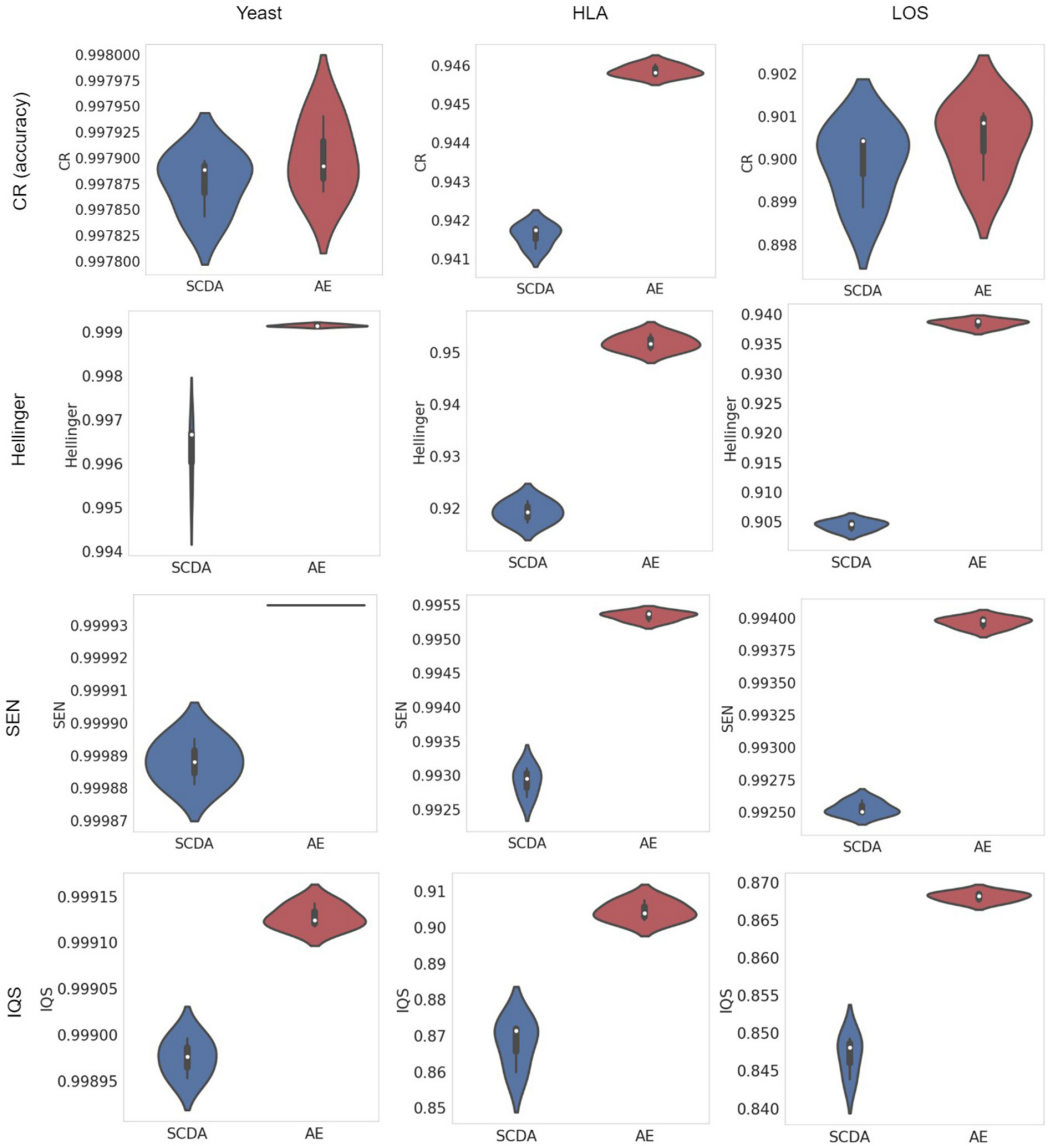
Violin plots of metrics (including the CR, the Hellinger score, the SEN score, and the IQS, from top to bottom) between the SCDA (blue color) and the AE (red color) model on the test datasets of yeast, HLA, and LOS data (from left to right) at the missing ratio of 20%.

A violin plot depicts not only the distribution of the numeric data (same as a box plot) but also its probability density. In other words, it shows summary statistics (e.g., median, interquartile range, and distribution except for outliers) and density of each variable (wider regions of a violin plot indicate values will occur more frequently, while narrower regions indicate values will occur less frequently). The results of evaluation metrics gathered three times between our AE model and the SCDA model on three different test datasets at the missing ratio of 20% are visualized in violin plots in Figure 5. We observed that our AE model had relatively higher metrics including the CR, the Hellinger score, the SEN score, and the IQS compared with the SCDA model.

#### Distribution of metrics with histogram

**Figure 6:**
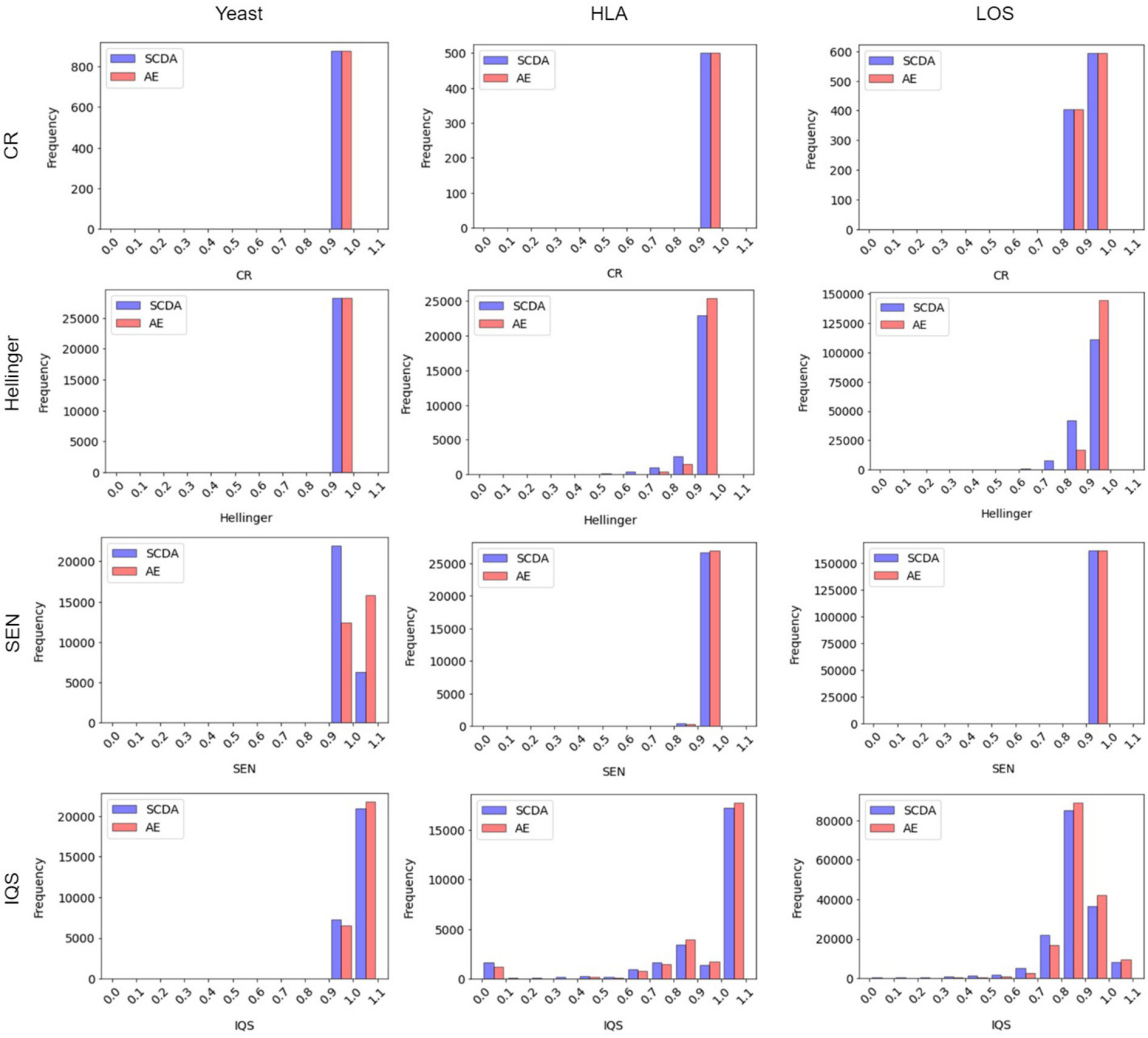
Histogram of metrics (including the CR, the Hellinger score, the SEN score, and the IQS, from top to bottom) between the SCDA (blue color) and the AE (red color) model on the test datasets of yeast, HLA, and LOS data (from left to right) at the missing ratio of 20%.

Figure 6 shows the frequency distribution of the metrics between our AE model and the SCDA model on three different test datasets at the missing ratio of 20%. We chose the histogram of the first run as an example because the results across all three runs were very similar. From this figure, we can see that for both the HLA and LOS data, our AE model achieved comparable distributions of the CR and SEN score to the SCDA model, while it had distributions closer to the right than the SCDA model (i.e., 1, indicating a higher imputation quality) for metrics including the Hellinger score and IQS. As for the yeast data, our AE model achieved comparable distributions of the CR and Hellinger score to the SCDA model, whereas it had the distributions closer to the right (i.e., 1) for metrics such as the SEN score and IQS compared with the SCDA model.

### Imputation quality with different MAFs

Table 6 shows the performance comparison in terms of evaluation metrics including the CR, the PCC, the Hellinger score, the minimum of the Hellinger score, the SEN score, the minimum of the SEN score, and the IQS between the AE and SCDA models on the test dataset of LOS data with four ranges of MAFs (e.g., MAF > 5%, 1% < MAF < 5%, 0.5% < MAF < 1%, and 0.1% < MAF < 0.5%) at a missing ratio of 20%. The AE model achieved overall better or comparable performance than the SCDA model in all four different ranges of MAFs, especially for the range of 0.1% <MAF <0.5% where our model demonstrated a considerably better IQS value (0.8767) than that of the SCDA model (0.0042). Based on the paired sample *t*-test, our model significantly outperformed the SCDA model in most scenarios.

**Table 6:** Performance results (mean, SD, and *p*-value with respect to different evaluation metrics) between the AE and the SCDA model on the test dataset of LOS data with different MAFs at the missing ratio of 20%.

**Metrics**	**Model**	**MAF**							
		**0.1% **< **MAF **< **0.5%**		**0.5% **< **MAF **< **1%**		**1% **< **MAF **< **5%**		**MAF** > **5%**	
		**mean (SD)**	***p*** **-value**	**mean (SD)**	***p*** **-value**	**mean (SD)**	***p*** **-value**	**mean (SD)**	***p*** **-value**
CR (accuracy)	SCDA	0.9931 (0.0002)	0.6730	0.9854 (0.0001)	0.0063	0.9544 (0.0011)	0.0701	0.7371 (0.0011)	0.0028
	AE	0.9932 (0.0001)		0.9859 (0.0)		0.9560 (0.0005)		0.7422 (0.0009)	
PCC (dosage)	SCDA	0.5901 (0.0108)	0.0006	0.7682 (0.0945)	0.1907	0.8334 (0.0021)	0.0005	0.8872 (0.0003)	0.0003
	AE	0.8915 (0.0002)		0.8980 (0.0003)		0.9011 (0.0002)		0.8987 (0.0002)	
Hellinger score	SCDA	0.9763 (0.0023)	0.0132	0.9624 (0.0010)	0.0064	0.9296 (0.0072)	0.0205	0.8472 (0.0029)	0.0011
	AE	0.9876 (0.0005)		0.9827 (0.0013)		0.9646 (0.0010)		0.8867 (0.0024)	
Minimum Hellinger score	SCDA	0.1336 (0.0067)	0.0023	0.0871 (0.0032)	0.0096	0.0670 (0.0070)	0.0169	0.1239 (0.0015)	0.0168
	AE	0.2572 (0.0047)		0.1255 (0.0043)		0.1152 (0.0034)		0.1420 (0.002)	
SEN score	SCDA	0.9988 (0.0)	0.0008	0.9987 (0.0002)	0.0285	0.9961 (0.0001)	0.0015	0.9845 (0.0)	0.0017
	AE	0.9997 (0.0)		0.9993 (0.0)		0.9979 (0.0)		0.9864 (0.0001)	
Minimum SEN score	SCDA	0.7760 (0.0023)	0.0014	0.7045 (0.0104)	0.0126	0.5908 (0.0199)	0.0439	0.3789 (0.013)	0.1708
	AE	0.8207 (0.0010)		0.7664 (0.0013)		0.6614 (0.0035)		0.4089 (0.0070)	
IQS	SCDA	0.0042 (0.0017)	0.0	0.7280 (0.0614)	0.0689	0.7848 (0.0105)	0.0056	0.8551 (0.0007)	0.0013
	AE	0.8767 (0.0008)		0.8844 (0.0002)		0.8836 (0.0001)		0.8699 (0.0004)	

We also observed two opposite trends of evaluation metrics for both our AE model and the SCDA model. With the increasing MAF, some metrics, including the CR, the Hellinger score, and the SEN score, declined. On the contrary, the PCC and IQS of the SCDA model increased when MAF was increased. This phenomenon is consistent with previous studies (Buckley et al., 2022; Kai-li et al., 2022). This is because the CR does not consider the correct genotype imputation with a random guess, especially for the rare variants. When MAF is increased, the probability of correct genotype imputation by chance decreased. On the other hand, the PCC is less sensitive to MAFs and the IQS adjusts for chance agreement and controls for allele frequencies. Therefore, both the PCC and MAF are more useful for the evaluation of imputation performance for rare variants. Interestingly, the PCC and IQS of our AE model have not shown a large difference for the four different ranges of MAFs, which means that our AE model is more robust to the impact of MAF in terms of the PCC and IQS metrics. As the LOS dataset is a multiethnic cohort including both Caucasian and African American samples, one of the advantages of our AE model compared with the SCDA model is that it can enhance the PCC and IQS imputation performance for rare variants, especially for African American data, which have more complicated genome structures and more rare variants.

## Discussion

In summary, we implemented a 1D convolutional AE model for genotype imputation and increased the imputation performance by improving the learning process. The evaluation results on the three genotype datasets revealed that our AE model achieved better (or at least comparable) imputation performance measured with metrics including the CR, the Hellinger score, the minimum of the Hellinger score, the SEN score, the minimum of the SEN score, and the IQS when compared with the reported SCDA model.

As our AE imputation is a reference-free genotype imputation method, we did not compare our model with reference-based methods such as IMPUTE5, BEAGLE5, and Minimac4. However, we did compare it with the reference-free genotype imputation SCDA model. For the other basic reference-free methods including average, KNN, and SVD, Chen and Shi (Chen and Shi, 2019) have already made a comprehensive investigation between their proposed SCDA model and these popular imputation methods, and the comparison results showed that the SCDA model achieved better imputation accuracy than these popular methods. Therefore, we did not include them for the comparison with our AE approach.

In the comparison of imputation performance between our AE model and the SCDA model, we used the same parameters in the model structure (such as number of epochs, fake missing ratio, batch size, the learning rate, L1 regularization, dropout rate, number of filters, kernel size of the 1D convolution window, pooling size, and random seed for data splitting) except for the training strategies which were different. In other words, we implemented a customized training loop in our AE model and improved the training process by using a single batch loss rather than the average loss over batches used by the SCDA model. As shown in Figure 4, the losses decrease smoothly for all cases with insignificant fluctuations. We found that minimizing the losses corresponding to two batches separately is more effective than minimizing the average loss over two batches because the minimization of the loss for the second batch is based on the loss that has already been minimized for the first batch. Therefore, the improved imputation performance mainly resulted from the model subclassing method of the training process implemented in our proposed AE model.

There are several limitations for our AE model. First, compared with reference-based imputation methods, which can impute small sample size from a large reference panel, our AE model may not be able to handle the imputation of small sample sizes as effectively since it needs more data to train the model sufficiently. Second, since our AE model does not require a reference panel, it lacks the ability to utilize key genetic characteristics such as mutations, linkage patterns, and recombination hotspots in the reference panels of hundreds of thousands of individuals. Lastly, the imputation accuracy can be affected by several factors such as sample size, sequencing coverage, population structure, and MAF. In our current study, we only considered the effect of different MAFs on imputation accuracy and did not investigate the impacts of other factors.

For the future work, based the imputed genotype data from the AE model, we will further perform downstream analyses on LOS data for GWAS, such as identifying novel causal variants in a fine-mapping study to verify the power of genotype imputation. In addition, to overcome the drawback of lack of the interpretability of the DL methods, we will integrate prior biological information into our DL model and define biologically plausible connections within the architecture of a deep neural network. Another interesting direction of genotype imputation is the imputation for low-coverage (e.g., 1 × coverage or less) WGS data, which can be seen as an alternative approach to SNP arrays. Methods for low-coverage WGS imputation include GLIMPSE (Rubinacci et al., 2021), QUILT (Davies et al., 2021), and GeneImp (Spiliopoulou et al., 2017). All of these methods are based on large reference panels. Therefore, we will have the advantage to extend our reference-free AE model to low-coverage WGS imputation.

## Conclusions

To address the problem of missing values in genotype data with deep learning methods, we implemented a convolutional AE imputation model with an improved learning strategy by using a single batch loss rather than the average loss over batches. We first evaluated our AE model with two public genotype datasets including the yeast data and the HLA data and then applied it to our own LOS data. Our modified AE imputation model outperformed the reported SCDA model in terms of the performance metrics CR, Hellinger score, SEN score, and IQS. Furthermore, our AE model significantly improved the IQS for rare variants, especially for the data from African Americans. We believe that our proposed method has a great potential to increase the statistical power of GWAS and enrich downstream GWAS analyses.

## Data availability statement

The yeast (https://github.com/work-hard-playharder/SCDA/tree/master/data) and HLA (https://www.internationalgenome.org/) data can be found online. The LOS data presented in this article is not readily available due to patient confidentiality. Requests to access to it should be directed to the figshare repository (https://figshare.com/) under a DOI: https://doi.org/10.6084/m9.figshare.21441078. The code of AE model is available from: https://github.com/mengsong28/Autoencoder_imputation.

## Ethics statement

The studies involving human participants were reviewed and approved by the Tulane University Institutional Review Board. The patients/participants provided their written informed consent to participate in this study.

## Author contributions

H-WD, CZ, and PG conceived and supervised this project. MS and JG designed and implemented the model and drafted the manuscript. XS provided the yeast and HLA data. ZL, CQ, LZ, K-JS, and QT contributed to the LOS data curation. CZ, HH, PG, H-WD, JL, and K-JS revised the first draft manuscript. JL, WZ, CW, HH, XS, and HS provided valuable insights into the original and revised manuscript. All authors have read and agreed to the final version of the manuscript for publication.

## Funding

This project was funded in part by grants from the U.S. National Institutes of Health (P20GM109036, R01AR069055, U19AG055373, R01AG061917, AR-27065, and M01 RR00585) and a grant awarded by the U.S. Engineer Research and Development Center (W912HZ20P0023).

## Conflict of interest

The authors declare that the research was conducted in the absence of any commercial or financial relationships that could be construed as a potential conflict of interest.

## Publisher's note

All claims expressed in this article are solely those of the authors and do not necessarily represent those of their affiliated organizations, or those of the publisher, the editors and the reviewers. Any product that may be evaluated in this article, or claim that may be made by its manufacturer, is not guaranteed or endorsed by the publisher.

## Author disclaimer

This article reflects the views of the authors and does not necessarily reflect those of the U.S. Food and Drug Administration and U.S. Army Corps of Engineers.
