# Supplementary material for: An autoencoder-based deep learning method for genotype imputation
Source: Front Artif Intell. 2022 Nov 3;5:1028978. doi: 10.3389/frai.2022.1028978 (PMC9671213; doi:10.3389/frai.2022.1028978)
Supplement: Supplementary file 1 [file Table_1.docx]

**Supplementary Information for: An Autoencoder-Based Deep Learning Method for Genotype Imputation**

Meng Song^1^, Jonathan Greenbaum^2^, Joseph Luttrell IV^1^, Weihua Zhou^3^, Chong Wu^4^, Zhe Luo^2^, Chuan Qiu^2^, Lan Juan Zhao^2^, Kuan-Jui Su^2^, Qing Tian^2,^ Hui Shen^2^, Huixiao Hong^5^, Ping Gong^6^, Xinghua Shi^7^, Hong-Wen Deng^2*^, Chaoyang Zhang^1*^

^1^School of Computing Sciences and Computer Engineering, University of Southern Mississippi, Hattiesburg, MS, USA

^2^Tulane Center of Biomedical Informatics and Genomics, School of Medicine, Tulane University, New Orleans, LA, USA

^3^College of Computing, Michigan Technological University, Houghton, MI, USA

^4^Department of Biostatistics, The University of Texas MD Anderson Cancer Center, Houston, TX, USA

^5^Division of Bioinformatics and Biostatistics, National Center for Toxicological Research, US Food and Drug Administration, Jefferson, AR 72079, USA

^6^Environmental Laboratory, U.S. Army Engineer Research and Development Center, Vicksburg, MS, USA

^7^Department of Computer & Information Sciences, Temple University, Philadelphia, PA 19122, USA

**Authors for correspondence:**
Dr. Chaoyang Zhang, [chaoyang.zhang@usm.edu](mailto:chaoyang.zhang@usm.eduDr) , Dr. Hong-Wen Deng, [hdeng2@tulane.edu](mailto:hdeng2@tulane.edu)

**Disclaimer:** This article reflects the views of the authors and does not necessarily reflect those of the U.S. Food and Drug Administration and U.S. Army Corps of Engineers.

1. **Supplementary for Evaluation Metrics**

We evaluated our model in terms of evaluation metrics including the concordance rate (CR), the Hellinger score, the scaled Euclidean norm (SEN) score, and the imputation quality score (IQS) (Stahl et al., 2021). In the following equations for different metrics, $x$ is the row vector of the original values, $y$ is the corresponding row vector of the imputed values, and $N$ is the number of samples.

CR is the ratio of correctly imputed SNPs out of all SNPs (Stahl et al., 2021):

$CR=\frac{M}{G}$⑴

where M is the number of matching SNPs, and G represents the total number of SNPs.

Hellinger score is a measure of the distance between two probability distributions (Roshyara et al., 2014):

$H\left( g \right)=1-\sqrt{1-\sum_{i=1}^{3} \sqrt{f_{x}^{i}(g)f_{y}^{i}(g)}}$⑵

where $f_{x}(g)$ and $f_{y}(g)$ are the trinomial probability distributions of the true genotype at g and the imputed genotype for each sample, respectively. The original true genotype probability of $f_{x}\left( g \right)$ can be defined as

$f_{x}(g))=(p_{0}, p_{1}, p_{2})=(p_{0}, p_{1}, 1-(p_{0}+p_{1}) )$⑶

where

$\left( p_{0}, p_{1}, 1-(p_{0}+p_{1}) \right)=\left\{ \begin{aligned} \left( \left( 1-\varepsilon\right)^{2}, 2\varepsilon\left( 1-\varepsilon\right), \varepsilon^{2} \right) if g=0 \\ \left( \varepsilon\left( 1-\varepsilon\right), \left( 1-\varepsilon\right)^{2}+\varepsilon^{2}, \varepsilon\left( 1-\varepsilon\right) \right) if g=1 \\ \left( \varepsilon^{2}, 2\varepsilon\left( 1-\varepsilon\right), \left( 1-\varepsilon\right)^{2} \right) if g=2 \end{aligned} \right.$⑷

and parameter $\varepsilon$ is the probability of genotyping errors ($\varepsilon=0$).

Similarly, the imputed true genotype probability of $f_{y}(g)$ can be defined as:

$f_{y}(g))=(\tilde{p_{0}}, \tilde{p_{1}}, \tilde{p_{2}})=(\tilde{p_{0}}, \tilde{p_{1}}, 1-(\tilde{p_{0}}+\tilde{p_{1}})$⑸

SEN score calculates the scaled Euclidean distance between the imputed dosage and true dosage per SNP per sample (Roshyara et al., 2014):

$S=(1-\frac{{(M^{obs}-M^{imp})}^{2}}{4})$⑹

where $M^{obs}$ and $M^{imp}$ are defined as follows:

$M^{obs}=0*p_{0}+1*p_{1}+2*\left( 1-\left( p_{0}+p_{1} \right) \right)=2-(2p_{0}+p_{1})$⑺

$M^{imp}=0*\tilde{p_{0}}+1*\tilde{p_{1}}+2*\left( 1-\left( \tilde{p_{0}}+\tilde{p_{1}} \right) \right)=2-(2\tilde{p_{0}}+\tilde{p_{1}})$⑻

IQS adjusts for chance agreement to assess the imputation accuracy with an emphasis on rare variants and is calculated based on the observed proportion of agreement ($P_{o}$) and the chance agreement ($P_{c}$) as follows (Lin et al., 2010):

$IQS=\frac{P_{o}-P_{c}}{1-P_{c}}$⑼

where $P_{o}$ and $P_{c}$are defined as:

$P_{o}=\frac{\sum_{i} n_{ii}}{n_{..}}$⑽

$P_{c}=\frac{\sum_{i} n_{i.}*n_{.i}}{{n_{..}}^{2}}$⑾

For each SNP across N samples, each cell of $n_{ij}$ in a $3\times3$ table (e.g., imputed genotypes for AA, AB, and BB, and true genotypes for AA, AB, and BB) represents the number of samples with imputed genotype i and true genotype j, and $n_{i.}$ and $n_{.i}$ are the marginal frequencies that would occur if genotypes are called randomly with the same marginal rates.

**Reference**

Browning, B. L., and Browning, S. R. (2009). A Unified Approach to Genotype Imputation and Haplotype-Phase Inference for Large Data Sets of Trios and Unrelated Individuals. *Am J Hum Genet* 84, 210–223. doi: 10.1016/j.ajhg.2009.01.005.

Lin, P., Hartz, S. M., Zhang, Z., Saccone, S. F., Wang, J., Tischfield, J. A., et al. (2010). A New Statistic to Evaluate Imputation Reliability. *PLOS ONE* 5, e9697. doi: 10.1371/journal.pone.0009697.

Roshyara, N. R., Kirsten, H., Horn, K., Ahnert, P., and Scholz, M. (2014). Impact of pre-imputation SNP-filtering on genotype imputation results. *BMC Genetics* 15, 88. doi: 10.1186/s12863-014-0088-5.

Stahl, K., Gola, D., and König, I. R. (2021). Assessment of Imputation Quality: Comparison of Phasing and Imputation Algorithms in Real Data. *Frontiers in Genetics* 12. Available at: https://www.frontiersin.org/articles/10.3389/fgene.2021.724037 [Accessed July 24, 2022].
